# Eco-friendly stability-indicating RP-HPTLC method for sildenafil analysis, characterization and biological evaluation of its oxidized stress degradation product

**DOI:** 10.1038/s41598-021-94854-6

**Published:** 2021-07-28

**Authors:** Maged S. Abdel-Kader, Prawez Alam, Gamal A. Soliman, Ramadan Al-Shdefat, Obaid Afzal

**Affiliations:** 1 Department of Pharmacognosy, College of Pharmacy, Prince Sattam Bin Abdulaziz University, P.O. Box 173, Al-Kharj, 11942 Saudi Arabia; 2 Department of Pharmacognosy, Faculty of Pharmacy, Alexandria University, Alexandria, 21215 Egypt; 3 Department of Pharmacology, College of Pharmacy, Prince Sattam Bin Abdulaziz University, P.O. Box 173, Al-Kharj, 11942 Saudi Arabia; 4 Department of Pharmacology, College of Veterinary Medicine, Cairo University, Giza, Egypt; 5 Department of Pharmaceutical Sciences, Faculty of Pharmacy, Jadara University, Irbid, Jordan; 6 Department of Pharmaceutical Chemistry, College of Pharmacy, Prince Sattam Bin Abdulaziz University, P.O. Box 173, Al-Kharj, 11942 Saudi Arabia

**Keywords:** Chemical biology, Chemistry

## Abstract

A feasible and cost effective reverse-phase high-performance thin layer chromatography (RP-HPTLC) based method was developed for the quantification of sildenafil (SLD) using eco-friendly EtOH:H_2_O (9.5:0.5 *v*/*v*) as mobile phase. SLD was subjected to stress conditions according to the International Conference on Harmonization (ICH) guidelines. The drug undergoes significant structural changes under oxidative stress condition to the N-oxide derivative. The oxidation product Sildenafil N-oxide (SDL N-oxide) designated in the European Pharmacopeia (EP) as impurity B was characterized utilizing 1D- and 2D-NMR as well as High Resolution Electrospray Ionization Mass Spectroscopy. The aphrodisiac potency of SDL N-oxide in comparison with SLD was evaluated in vivo using rats as experimental animal model. The evaluation based on the following parameters: mount, intromission and ejaculation latencies (ML, IL and EL, respectively), mounting and intromission frequencies (MF and IF, respectively), and postejaculatory interval (PEI). SLD N-oxide expressed similar aphrodisiac effect to SLD but with less potency. Molecular docking of SDL N-oxide along with the parent drug SLD, indicated a strong binding affinity and similar binding pattern within the active site of phosphodiesterase 5 (PDE5). However, the docking score of SLD N-oxide was slightly lower as compared to SLD in agreement with the biological study findings.

## Introduction

In 1989, sildenafil (SLD) was primarily investigated as medication for the treatment of angina pectoris and hypertension. However, during clinical trials, SLD manifested a little impact on angina pectoris and marked effect on penile erection. Consequently, in 1998, Pfizer repurposed and marketed SLD for erectile dysfunction^1^. SLD is a selective blocker of phosphodiesterase 5 (PDE5) preventing the degradation of cyclic guanosine monophosphate (cGMP). Increased levels of cGMP, induce vasodilation as one of the body smooth muscles. This vasodilatation cause increase in the blood flow into the spongy tissue of the penis leading to erection^2^. SLD is a repeatedly cited model of drug repurposing. Several drugs have been successfully repurposed in the near past such as minoxidil^3^. However, SLD is considered as one of the typical examples of drug repurposing^4^. Moreover, since 2005, SLD has been also approved by U.S. Food and Drug Administration (FDA), Europe, the Middle East, and Africa (EMEA) for treatment of pulmonary arterial hypertension^5^.

Several stability indicating HPLC studies were developed for the estimation of SLD^6–8^. However, none of these methods attempted to characterize the degradation products. Other sophisticated analytical techniques including HPLC, LC/MS/MS spectroscopy offers many advantages like rapidness, reliability and accuracy. However; HPTLC gives the unique advantages of simultaneous analysis of several samples using the same chromatogram. In addition, HPLTC utilizing a relatively very small quantity of the mobile phase. This makes HPTLC the most time and cost-effective method of chromatographic analysis^9,10^. HPTLC is also feasible for the development of chromatographic fingerprint to determine and identify complex herbal extracts just like HPLC and GC. Another advantage of HPTLC is the higher sensitivity and capability to detect more compounds than HPLC. The colourful pattern and quantification at the micron and nanogram levels helps to compare various samples on the same plate^11^^,^^12^.

Two HPTLC methods utilizing normal phase silica gel plates were developed for the quantification and detection SLD in tablets and herbal formulations adulterated with the drug^13,14^. Another HPTLC method was designed for the simultaneous quantification of marketed table formulation containing SLD and dapoxetine^15^. Dapoxetine is used in this formulation to control premature ejaculation (PE) in men^16^. The reported HPTLC methods had also utilized most of the toxic solvents in the mobile phase^17^. None of the different techniques reported for the quantification and detection of SLD in different pharmaceutical dosage forms attempt to use reverse-phase silica gel or the green analytical technique. In the recent years, analytical techniques associated with “green analytical chemistry or environmentally-benign analytical techniques” have been increased significantly in literature^18,19^.

## Materials and methods

**Figure 1:**
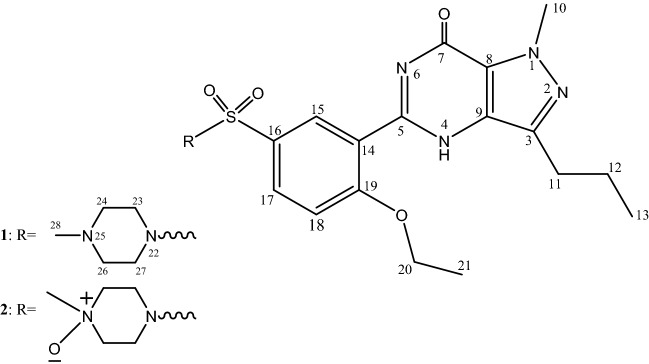
Structures of Sildenafil (SLD) and Sildenafil N-oxide (SLD N-oxide).

The following Standards were used in the study: sildenafil citrate (SLD) (Purity > 99%) (Fig. 1) (Sigma-Aldrich, St. Louis, MO, USA), SLD (Viagra) (Pfizer Inc, USA), hydroxyprogesterone (Purity ≥ 99%) (Bayer Pharma AG, Germany) and oestradiol benzoate (Purity ≥ 98%) (Misr Co. for Pharm. Ind., Egypt). Four marketed products (Film coated Tablet, 50 mg) approved in Saudi Arabia were obtained from Drug stores in Riyadh. Chromatographic and analytical grade reagent (AR) were used for extraction and method development. Pre-coated, glass-baked TLC plates obtained from E. Merck (RP-18-60F254; thickness: 0.2 mm; area: 20 × 10 cm) were used for quantification.

### Preparation of the sample and standard solution

Stock solution of SLD at concentration of 10 mg/ml was prepared in HPLC grade ethanol using 10 ml volumetric flask. The prepared solution was further diluted to obtaine 100 μg/ml solution used as a final stock solution. The average weights of four marketed formulations (Film coated Tablet; 50 mg) were calculated, crushed into fine powders and weights equivalent to the average weights were separately extracted with ethanol. The extracts were filtered to remove any undissolved materials using Whatman filter paper no. 4. The resulted clear solutions were separately diluted to100 μg/ml and kept at 4 °C for further analyses.

### Chromatographic conditions

TLC glass plates, size 10 × 20 cm (RP-18,60 F254) were used for the estimation of SDL. Samples were applied on the TLC plates using Camag microlitre syringe. The band width was set at 6 mm with the help of TLC auto sampler (ATS4).

**Figure 2:**
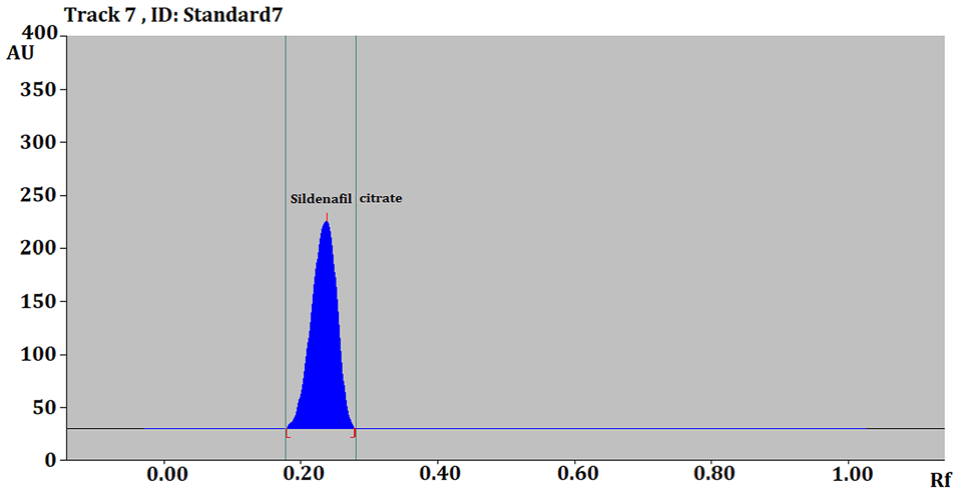
HPTLC Chromatogram of standard SLD.

The sample injections rate was set at 150 nl/s. The plates were developed with linear ascending order using eco-friendly mobile phase composed of EtOH:H_2_O (9.5:0.5 *v*/*v*) in Camag Automatic Developing Chamber 2 (ADC2) to the distance of 80 mm. The Chamber was saturated with the mobile phase vapour for 30 min at 22 °C prior to the development of the plates. After development, the dried TLC plates were scanned at 315 nm using Camag TLC scanner assembled with deuterium lamp system in absorbance mode (Fig. 2). Scanning parameters were adjusted to 4 mm × 0.1 mm slit dimension with speed of 20 mm/s.

Different concentrations of SLD citrate (100–700 ng/spot) were prepared by dilution of the standard stock solution. Different volumes (1–7 μl) were loaded on the TLC plates each in triplicate. After development of the plates, peak height, peak area versus concentration data were treated by linear regression analysis to obtain the calibration plot.

### Forced degradation of SLD

The obligatory deprivation of SLD densitometry methods was resulted under numerous stress circumstances like acid-induced, base-induced, oxidative, and photolytic degradation following the procedures described earlier^20,21^.

### Acid and base-induced degradation

Accurately weighed 10 mg SLD were dissolved in ethanol (5 ml) and mixed with either 1 M HCl or 1 M NH_4_OH in triplicates. The prepared solutions were kept at room temperature in dark place to ignore the possibility of photodegradation. The developed HPTLC method was applied for SLD quantification. Although no effect was observed after 24 h, in the acid medium SLD started to hydrolyze after 48 h and by the end of the 5th day no SLD could be detected in the solutions. No effect was observed in the alkaline solution.

### Hydrogen peroxide and temperature based degradation

**Figure 3:**
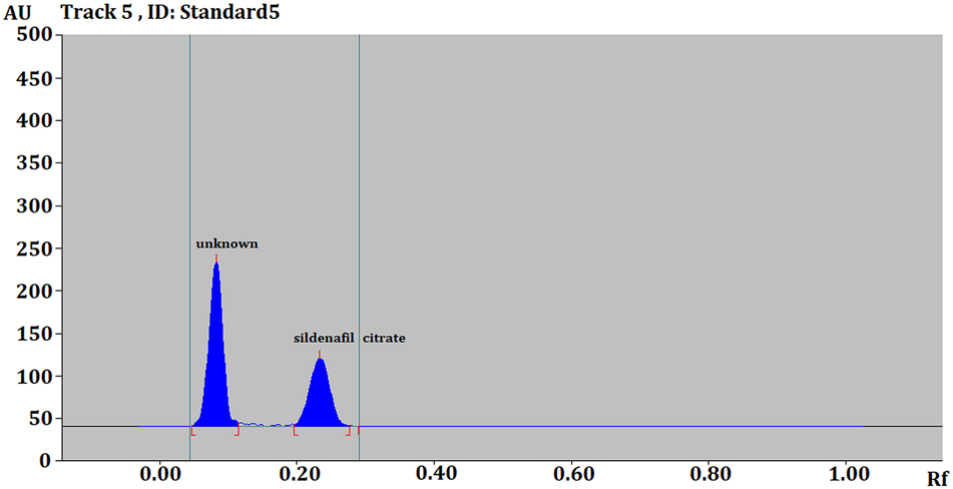
Chromatogram of hydrogen peroxide-induced degradation products of SLD at 315 nm wavelength.

Accurately weighed 2 mg/ml SLD was prepared in ethanol and treated with hydrogen peroxide (30.0%, v/v) in conical flasks in triplicate. The reaction mixtures were reserved at room temperature under dark regime to avoid possibility of photodegradation. The experiment was observed for 7 days. Degradation started after day one and the amount of SLD decreases by the time with the appearance of another peak along with the SLD by the end of the 7th day (Fig. 3). Similarly, temperature forced degradation was explored. Seven samples were separately dissolved in ethanol and kept in oven at 45 °C in well closed vials, while another seven samples were store at 55 °C. The SLD contents in each sample was measured on daily basis. The concentrations of SLD were calculated using the developed quantifications methods.

### Method validation

The linearity of SLD was measured using standard concentration range i.e.100–700 ng/spot. The standard curve for SLD was plotted between concentration (X-axis) and peak area (Y axis) under Least square linear regression analysis.

The pre-analyzed sample of SLD (200 ng/spot) was enriched with 0, 50, 100 and 150% SLD standard and the resulted solutions were re-quantified in triplicates. The % recovery and percent relative standard deviation (% RSD) were taken as measures for the method’s accuracy.

The precision of the method was measured on the basis of repeatability and intermediate precision. The repeatability and intermediate precision were assessed as intra-day precision and difference at 300, 400 and 500 ng/spot concentration levels each in triplicates.

Thoughtful variations in the mobile phase during analysis of SLD were noted as an indication for the method robustness.

Further, the method sensitivity (LOD) and (LOQ) were analyzed based on standard deviation using the formula below:

$${\text{LOD}} = 3.3 \times {\text{SD}}/{\text{S}};\quad {\text{LOQ}} = 10 \times {\text{SD}}/{\text{S}}$$

### Quantification of SLD in tablets

The test samples of each marketed product were resolved on the TLC plates and densitograms were obtained in identical circumstances of standard SLD. The R_f_ value of SLD was verified in the test samples and the concentrations of SLD were deliberated from the linear regression equation.

### Isolation of H_2_O_***2***_ degradation product

The oxidation product was chromatographically purified on silica gel column (50 gm x 1 cm i.d.). Elution started with chloroform followed by chloroform and methanol mixtures in a gradient system. Thirty-five fractions 25 ml each were collected, screened by TLC and similar fractions were pooled. Fractions 9–15 showed single spot with R_f_ value similar to SLD standard. Fractions 18–26 showed single spot with lower R_f_ value representing the degradation product.

### Spectral analysis

The UV spectrum was measured using a Unicum Heyios UV–visible spectrophotometer. ^1^H, ^13^C-NMR and 2D-NMR data were collected using Bruker UltraShield Plus 500 MHz spectrometer located at the College of Pharmacy, Prince Sattam Bin Abdulaziz as described earlier^22^. HRESIMS were determined using Thermo Scientific UPLC RS Ultimate 3000—Q Exactive hybrid quadrupole-Orbitrap mass spectrometer fitted with high performance quadrupole precursor selection with high resolution, accurate-mass (HR/AM) Orbitrap™ detection^23^.

### Ethical standards

The Bioethical Research Committee (BERC) at Prince Sattam Bin Abdulaziz University under IRB No. BERC-002-12-19 approved the experimental procedures. The study was carried out in compliance with the ARRIVE guidelines.

## Sexual behavior testing protocol

### Ethics statement

The Committee of Bioethical Research at Prince Sattam Bin Abdulaziz University under IRB No. BERC-002-12-19 approved the investigational plan. The study was carried out in compliance with the ARRIVE guidelines and following all the relevant guidelines. None of the used rats died or was euthanized because of this study.

### Animals

Eighteen male (220–250 g b.wt) and eighteen female (150–160 g b.wt) Wistar rats were obtained from the Animal Unit at Prince Sattam bin Abdulaziz University, Al-Kharj, Kingdom of Saudi Arabia. Each animal was kept in a separate cage under controlled conditions of temperature (22 ± 1 °C), 12 h dark/light cycle and relative humidity of 55 ± 10%. Standard rat feed and tap water were available ad libitum.

### Preparation of animals

Sexual training was encouraged by pairing each male to an oestrous female rat three times a week for 3 successive weeks. Only males displaying sexual activity were chosen for the experiment. Female animals were made responsive by subcutaneous administration of estradiol benzoate at 10 mg/kg b.wt and hydroxyprogesterone at 1.5 mg/kg b.wt, 48 and 4 h prior to coupling, respectively. Sexual capability of female rats was confirmed by pairing them with male rats. The most sexually active female animals were chosen for the experiment.

### Copulatory behaviour testing^24^

Three groups of sexually active male rats, 6 animals each, were chosen for the study. Each male rat was placed in an individual cage. Animals of the 1st group received a single oral dose of the vehicle (distilled water) at 10 ml/kg b.wt and served as normal controls (NC). Animals of the 2nd group received SLD in distilled water at 10 mg/kg b.wt and served as positive controls. Rats of the 3rd group received SLD N-oxide in distilled water at 10 mg/kg b.wt. The test was carried out in a quiet room under faint red light after 4 h of hydroxyprogesterone administration to females and 30 min after SLD, SLD N-oxide administration to males. Each sexually active female rat was introduced to a male cage of 50 × 30x30 cm dimensions. Different parameters of the sexual behaviour of male rats were observed and persisted for the first two mating series.

### Sexual behaviour parameters

ML (Mounting latency) and IL (Intromission latency): Duration from the onset of pairing of male and female animals to the first mount and to the first intromission (vaginal penetration) by the male, respectively.

MF (Mount frequency) and IF (Intromission frequency): Number of mounts and number of intromission (vaginal penetration) prior to ejaculation, respectively.

EL (Ejaculation latency): Time interval between first intromission and ejaculation. In the second mating series only the ejaculation latency was calculated.

PEI (Postejaculatory interval): Duration between ejaculation and first intromission of subsequent copulatory series.

Ejaculation in rats is recognized by a long, deep thrust (750–2000 ms) and much slow disjoint^25^. Next to ejaculation, the rat licks its fur, grooms with the forepaws and then snoozes during PEI that may persist for 10 min prior to restarting mating.

Depending on sexual behaviour parameters, copulatory and intercopulatory efficiencies (CE and ICE, respectively) can be computed^26^.

$$\begin{aligned} {\text{CE}} & = \left( {{\text{IF}}/{\text{MF}}} \right) \times 100. \\ {\text{ICE}} & = \left[ {{\text{IF}}/\left( {{\text{MF}} + {\text{IF}}} \right)} \right] \times 100. \\ \end{aligned}$$

The experiment was disconnected when the male be unsuccessful to demonstrate sexual activity or when the female did not show sexual interest.

### Statistical analysis

The significance of difference between the means was determined by one-way analysis of variance (ANOVA) with post-hoc 't' test. P values of less than 0.05 were considered as statistically significant.

### Molecular docking

Computational studies were executed by Maestro program, version-10.5 (GUI of Schrodinger 2017) installed on a desktop. Molecular docking studies were carried out using Glide module implemented in Maestro by extra precision (XP) docking of ligands^27^. The PDB file of X-ray crystal structure of PDE5 in complex with SLD (PDB ID: 2H42, 2.30 Å) was downloaded from the website of RCSB PDB and utilized for the molecular docking studies^28^. The preparation of structure, corresponding to the removal of water, consignment of bond orders, insertion of H atoms, and dealing of formal charges were done by Protein Preparation Wizard in Maestro. The network of hydrogen bonds in the protein structure was optimized through exhaustive sampling facility. The energy was minimized to RMSD of 0.3 Å by utilizing Impref by using the force field OPLS_2005. A grid (20 × 20 × 20 Å) was created by describing the co-crystallized ligand. LigPrep with Epik module was utilized to expand protonation, and tautomeric states of ligands (SLD and SLD N-oxide) at 7.0 ± 2.0 pH. The energy of the protein structure was then minimized by the force field OPLS_2005. The molecular docking was performed by Glide XP docking.

## Results

### HPTLC analysis

**Figure 4:**
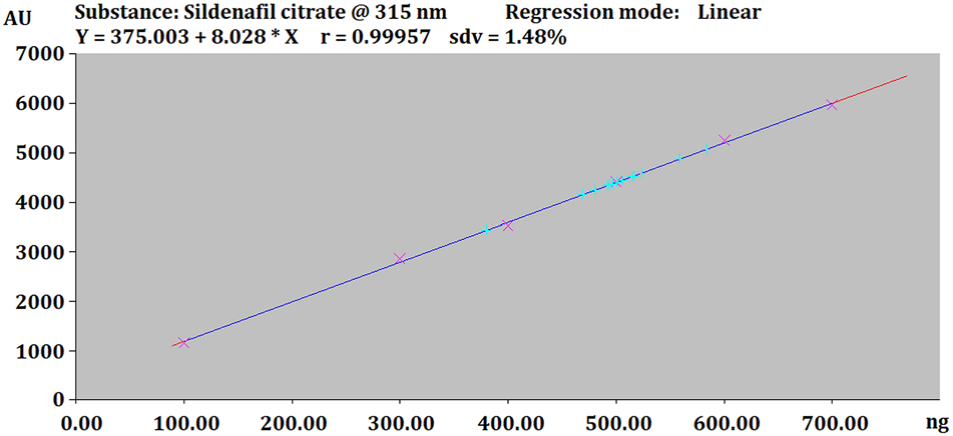
Linearity graph of SLD.

Calibration data and linearity are presented in Table 1, Figs. 2 and 4. Accuracy, precision and robustness data are presented in Tables 2, 3 and 4, respectively. Quantification of SLD in tablets is presented in Table 5.

**Table 1:** Linear regression data for the calibration curve of SLD (n = 6).

Linearity range (ng/spot)	100–700
Regression equation	Y = 7.8429x + 468.09
Correlation coefficient (R^2^)	0.9995
Slope ± SD	1.1722 ± 0.0529
Intercept ± SD	1657.5 ± 254.69
Standard error of slope	0.02162
Standard error of intercept	104
95% confidence interval of slope	1.113–1.234
95% confidence interval of intercept	960–1538

**Table 2:** Accuracy of the proposed method (n = 6).

Excess drug added to analyte (%)	Theoretical content (ng)	Conc. found (ng) ± SD	% Recovery	% RSD
0	200	195.33 ± 1.63	97.67	0.84
50	300	293.33 ± 3.20	97.78	1.09
100	400	397.00 ± 1.41	99.25	0.36
150	500	491.00 ± 4.60	98.20	0.44

**Table 3:** Precision of the proposed method.

Conc (ng/spot)	Repeatability (intraday precision)			Intermediate precision (interday)		
	Area ± SD (n = 6)	SE	% RSD	Area ± SD (n = 6)	SE	% RSD
300	2857.80 ± 23.20	9.47	0.81	2853.20 ± 30.58	12.49	1.07
400	3550.60 ± 28.50	11.64	0.80	3552.20 ± 31.72	12.95	0.89
500	4351.60 ± 33.00	13.06	0.74	4349.80 ± 34.78	14.20	0.80

**Table 4:** Robustness of the proposed HPTLC method.

Conc (ng/spot)	Mobile phase composition (methanol: water)			Results		
	Original	Used		Area ± SD (n = 6)	% RSD	R_f_
400	9:1	8.9:1.1	+ 0.1	3565 ± 27	0.75	0.24
		9:1	0.0	3551 ± 33	0.94	0.23
		9.1:0.9	− 0.1	3547 ± 39	1.09	0.21

**Table 5:** Quantification of *SLD* in marketed tablets (n = 3).

Samples	Theoretical content (mg)	Content (%)
GS	50	99.22
VS	50	99.31
FS	50	98.65
AS	50	99.01

### Structure elucidation of H_2_O_2_ degradant of SLD

**Figure 5:**
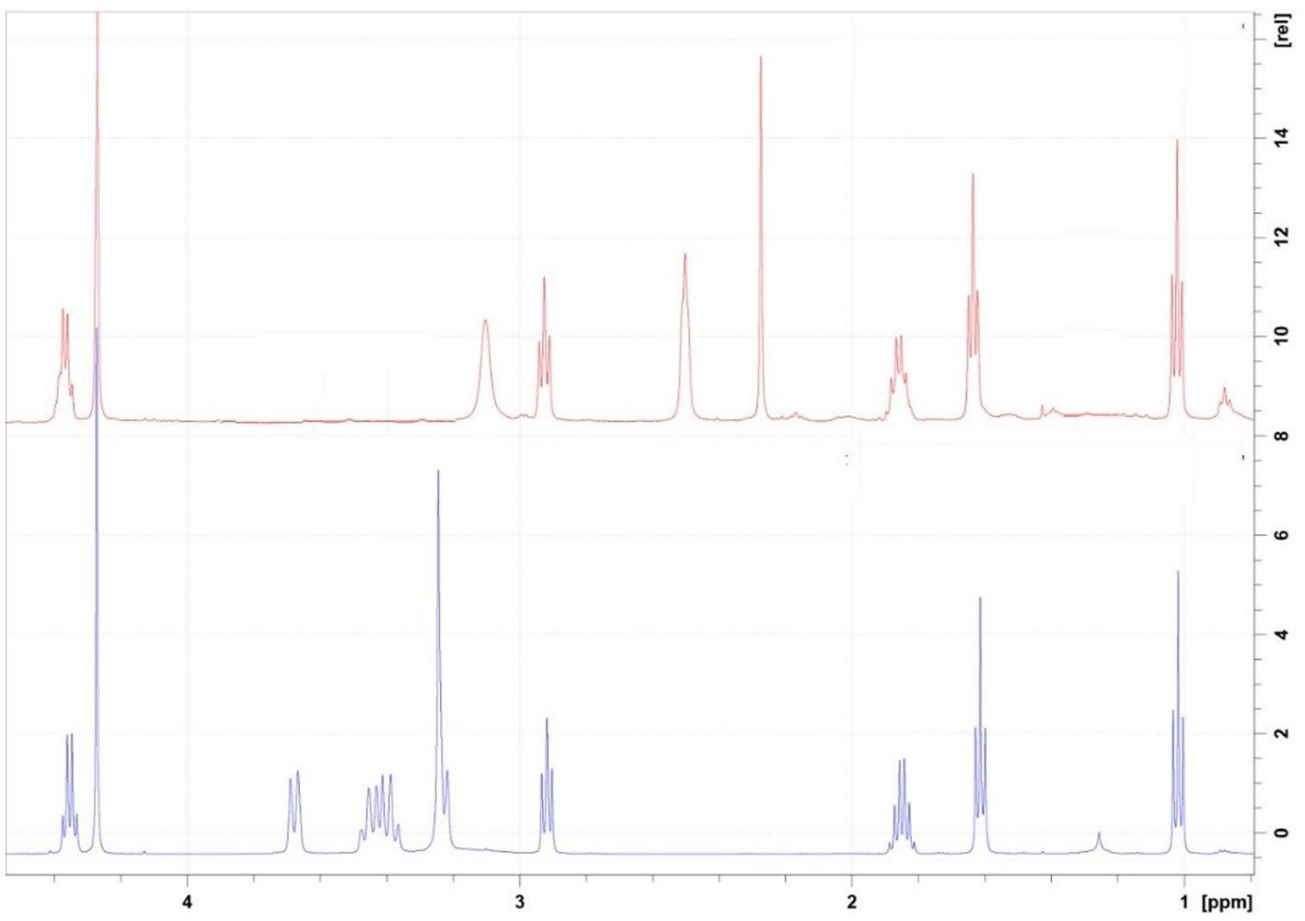
^1^H NMR major differences between Sildenafil (Upper Red) and Sildenafil N-oxide (lower Blue).

**Figure 6:**
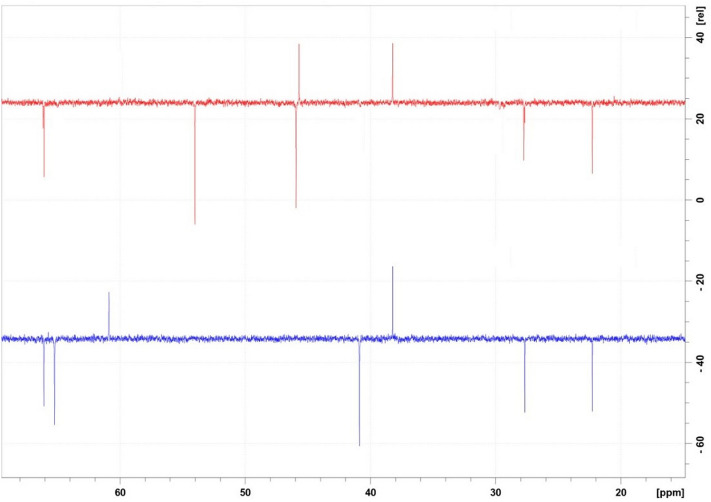
DEPT 135 major differences between Sildenafil (Upper Red) and Sildenafil N-oxide (lower Blue).

**Figure 7:**
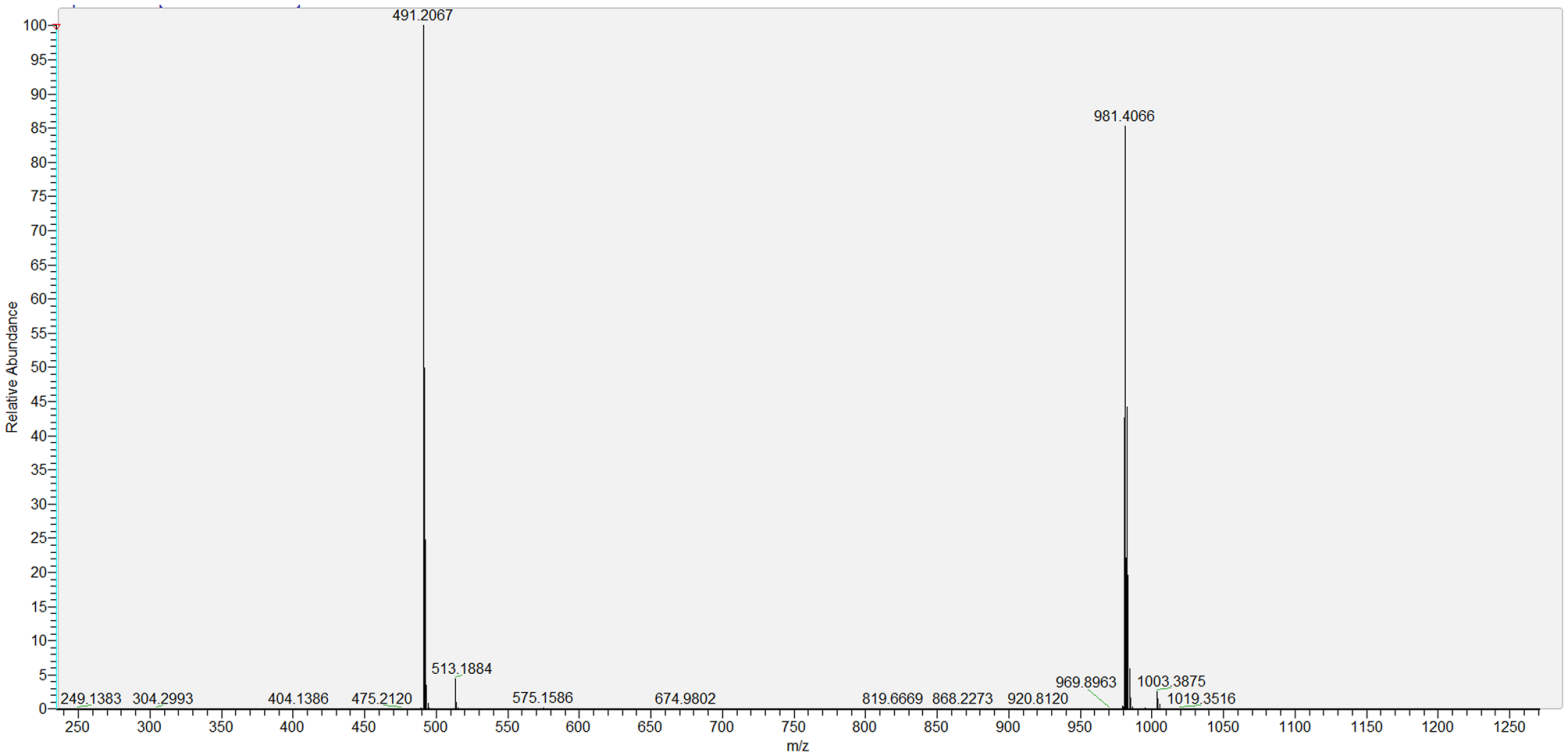
HRESIMS of Sildenafil N-oxide in the positive mode.

**Figure 8:**
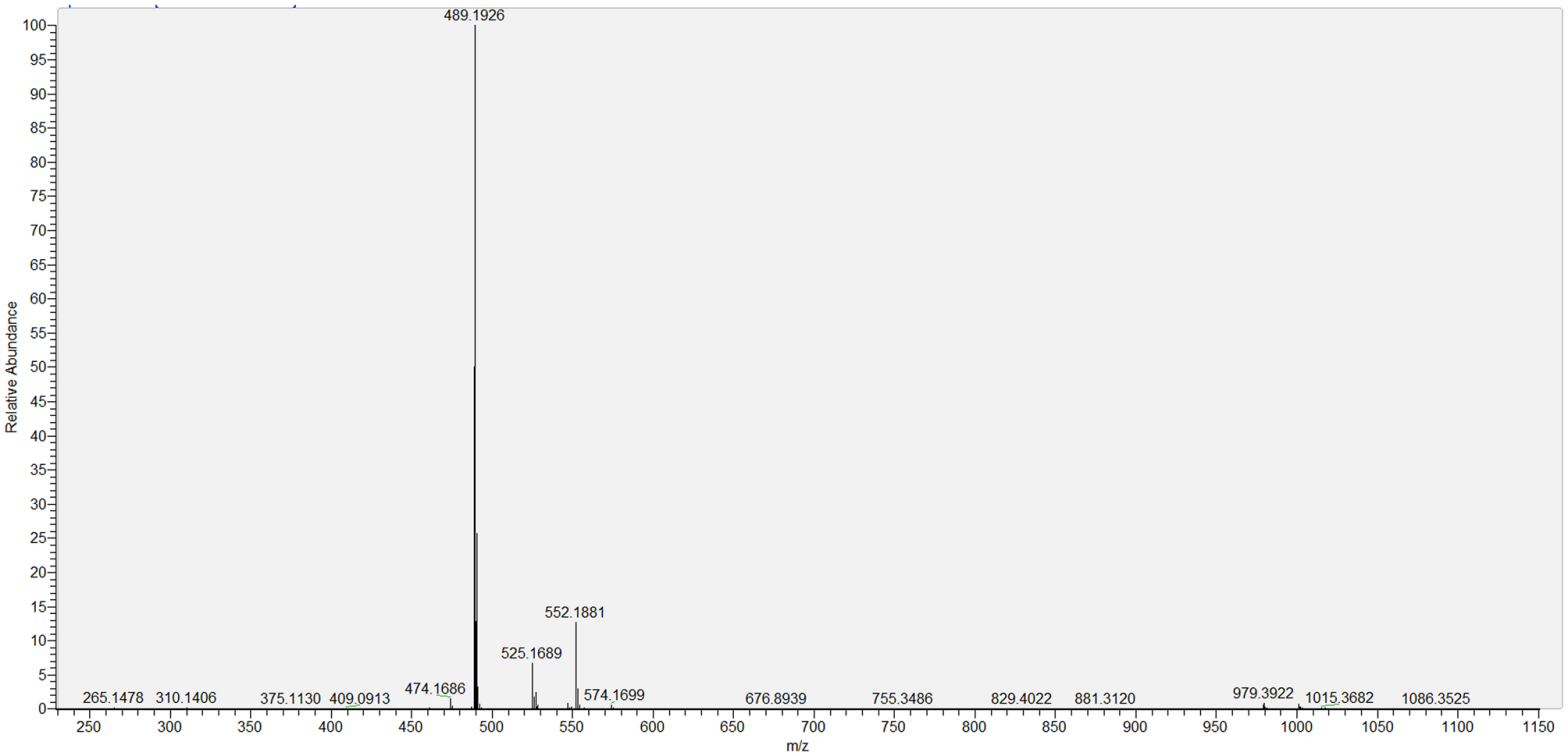
HRESIMS of Sildenafil N-oxide in the negative mode.

*Sildenafil N-oxide* C_22_H_30_N_6_O_5_S; White powder; UV λ_max_ MeOH: 237, 311 nm; IR (KBr) υ_max_ 3343, 1714, 1204, 918 cm^−1^; ^1^H and ^13^C NMR see Table 6, Figs. 5 and 6; HRESIMS: [2 M + 1]^+^ *m*/*z* 981.4066 (calcd for C_44_H_61_N_12_O_10_S_2_, 981.4075), [M + 1]^+^ *m*/*z* 491.2067 (calcd for C_22_H_31_N_6_O_5_S, 491.2077), [M-1]^+^ *m*/*z* 489.1926 (calcd for C_22_H_29_N_6_O_5_S, 489.1920) (Figs. 7,8).

**Table 6:** ^1^H-, ^13^C-NMR of SLD and SLD N-oxide in CDCl_3_*.

Pos	SLD		SLD N-oxide	
	^1^H**	^13^C	^1^H**	^13^C
3	–	137.93	–	138.29
5	–	153.75	–	153.93
7	–	146.96	–	146.76
8	–	124.52	–	124.56
9	–	145.02	–	146.64
10	4.27 (s)	38.15	4.23 (s)	38.19
11	2.93 (t, J = 7.6)	27.66	2.88 (t, J = 7.6)	27.62
12	1.86 (q = J = 7.4)	21.81	1.86 (m)	22.26
13	1.02 (t, J = 7.3)	14.10	0.99 (t, J = 7.4)	14.02
14	–	121.18	–	122.12
15	8.78 (d, J = 2.2)	131.20	8.60 (bs)	131.03
16	–	128.80	–	128.09
17	7.82 (dd, J = 2.2, 8.7)	131.70	7.78 (bd, J = 8.5)	131.40
18	7.15 (d, J = 8.7)	113.08	7.12 (d, J = 8.5)	113.35
19	–	160.01	–	159.96
20	4.36 (q J = 7.7)	64.93	4.29 (q, J = 7.5)	65.97
21	1.64 (t, 7.7)	14.44	1.55 (t, 7.5)	14.48
23, 27	3.10 (bs)	45.87	3.40 (q, J = 9.7) 3.63 (d, J = 14.7)	40.84
24, 26	2.50 (bs)	53.68	3.21*** 3.39 (t, J = 11.0)	65.07
28	2.27 (s)	45.64	3.20 (s)	60.61

*Assignments based on COSY, HSQC, HMBC, H2BC and comparison with literature data for SLD.

**δ ppm, *J* in parentheses in Hz.

***Overlapped signals.

### Sexual behavior testing

**Figure 9:**
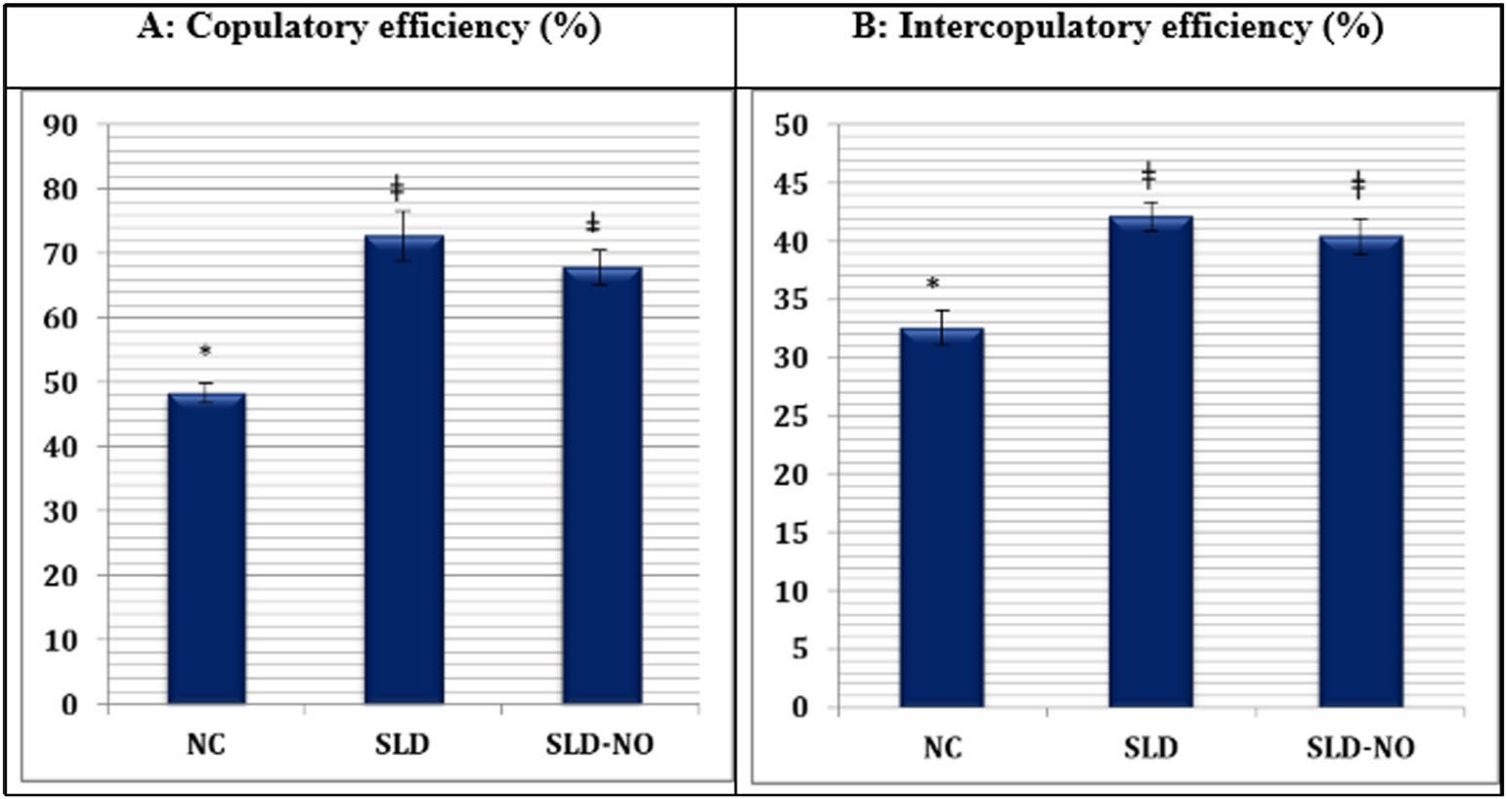
Effect of SLD and SLD N-oxide on the copulatory (**A**) and intercopulatory (**B**) efficiencies of male rats.

The aphrodisiac effects of SLD and SLD N-oxide are shown in Tables 7, 8 and Fig. 9.

**Table 7:** Effect of SLD and SLD N-oxide on mount latency and frequency and intromission latency and frequency of male rats (n = 6).

Treatment	Dose (mg/kg)	ML (s)	MF	IL (s)	IF
NC	00	117.35 ± 4.52*	6.62 ± 0.52*	221.47 ± 6.73*	3.20 ± 0.12*
SLD	10	44.17 ± 2.28ǂ	17.50 ± 0.98ǂ	87.21 ± 3.58ǂ	12.74 ± 0.78ǂ
SLD N-oxide	10	67.87 ± 2.73ǂ*	12.64 ± 0.69ǂ*	127.08 ± 3.74ǂ*	8.58 ± 0.44ǂ*

ǂIndicate significance in comparison to NC group at *p* < 0.05.

*Indicate significance in comparison to SLD group at *p* < 0.05.

**Table 8:** Effect of SLD and SLD N-oxide on ejaculation latency in 1st and 2nd series and post ejaculatory interval of male rats (n = 6).

Treatment	Dose (mg/kg)	EL-1 (s)	PEI (s)	EL-2 (s)
NC	00	362.27 ± 9.25*	454.26 ± 17.42*	382.56 ± 9.50*
SLD	10	457.52 ± 14.70ǂ	329.27 ± 10.26ǂ	484.28 ± 12.51ǂ
SLD N-oxide	10	405.71 ± 14.52ǂ*	396.70 ± 9.53ǂ*	434.37 ± 12.14ǂ*

ǂIndicate significance in comparison to NC group at *p* < 0.05.

*Indicate significance in comparison to SLD group at *p* < 0.05.

## Discussion

**Figure 10:**
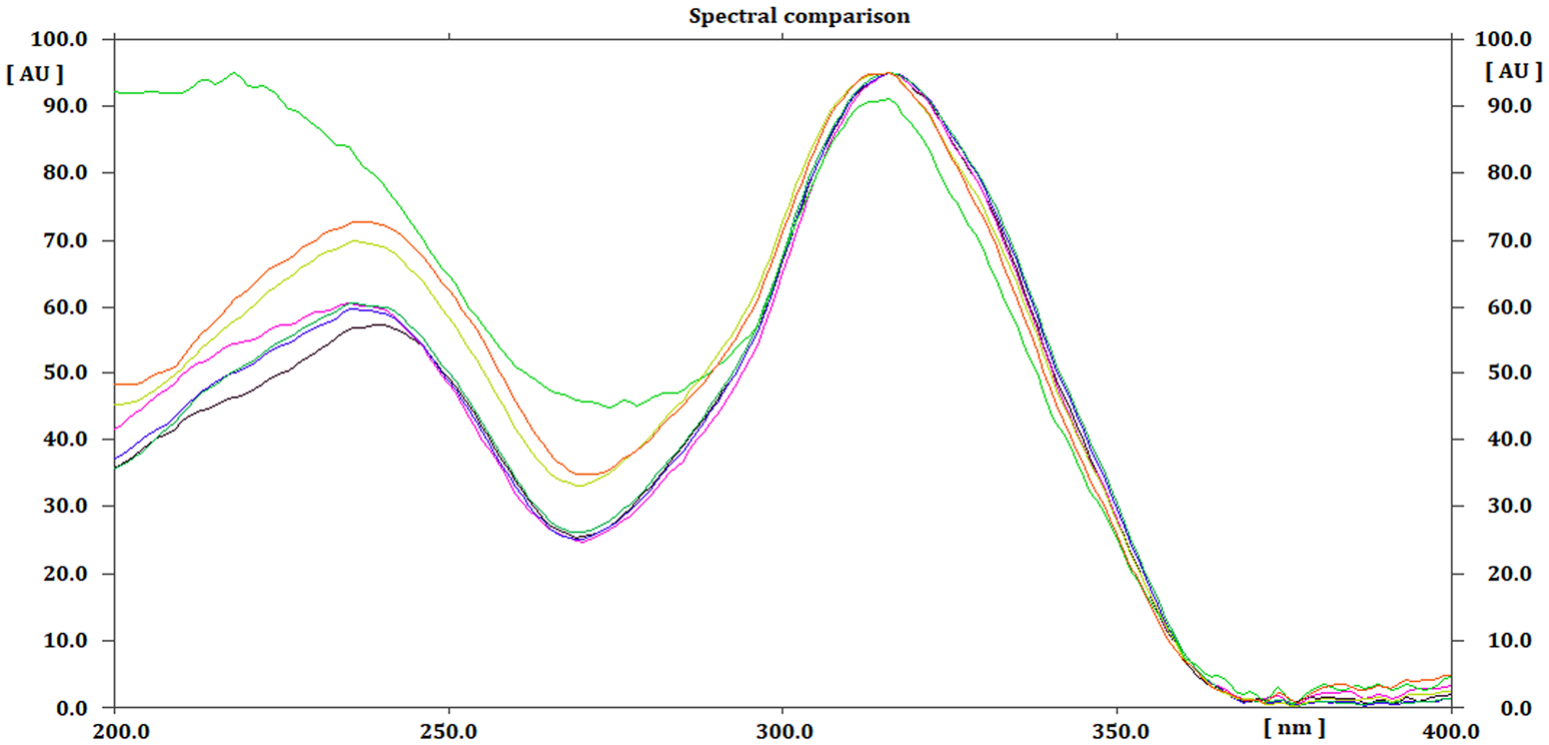
Overlay UV absorption spectra of standard SLD and acid, base, hydrogen peroxide and different temperature.

An eco-friendly HPTLC method was developed for the quantification of SLD. The proposed mobile phase composed of the green solvent mixture EtOH: H2O (9.5:0.5 *v*/*v*) produced sharp, symmetrical and well resolved peak at R_f_ value of 0. 23 (Fig. 2) for SLD standard after saturation for 30 min. The measured UV spectra SLD spots showed λ_max_ absorption at 315 nm. This wave length was chosen for UV densitometric analysis (Fig. 10).

The calibration curve correlating spot areas versus concentrations (ng/spot) expressed linearity between 100 and 700 ng/spot (Fig. 4) with valid linear regression data at the stated rang and highly significant correlation coefficient value (R^2^) (*p* < 0.05) (Table 1). No significant differences were observed in the slope of standard curves (*p* ≥ 0.05). The regression equation was Y = 7.8429X + 468.09 with correlation coefficient (R^2^) of 0.9995 (Table 1). The method’s accuracy was proved by recovery analysis expressed as 97.67–99.25% recovery after spiking previously analyzed test solution with standard SLD. The low values of % RSD (0.36–1.09) indicated the high accuracy of the method (Table 2). Results of repeatability (intraday precision) and intermediate precision (Interday precision) were expressed as % RSD (Table 3). The very low values (0.75–1.09) of % RSD gave an indication about the good repeatability and intermediate precision of the proposed HPTLC method. The robustness of the methods was explored by slight changes in the mobile phase polarity for the sample with analyte’s concentration of 200 ng/spot (Table 4). Low values of % RSD (≤ 2) obtained after the induced changes proved the robustness of the method. LOD and LOQ were 41.12 and 125.36 ng/spot respectively, indicating a wide range of effective quantification of SLD can be achieved applying the proposed method.

The peaks of SLD in sample solutions were concluded from their R_f_ values compared with standard SLD. Tablets analyzed under same HPTLC conditions applied for standard SLD showed spots at same R_f_ value (0.23) as that of the standard. The obtained SLD percentage in the four analyzed samples were 97, 98, 96 and 95 in A, B, C, and D samples respectively (Table 5).

The developed method encouraging resolution of SDL standard as well as in different pharmaceutical products. The recovery of nearly 100% indicated no peak interference among the standard compound and other components of the formulations. HPTLC chromatogram indicated that the samples and standards were separated without diffusing and/or tailing.

SLD expressed high degree of stability against thermal degradation as well as modest alkaline medium using NH_4_OH solution. However, considerable loss of the drug was gradually recorded in acid medium resulting in complete loss of SLD by the day 5. This degradation most likely targeted the pyrimidine-4(*1H*)-one ring containing the labile amide (=N–C=O) function. Similarly, SLD undergo structural change under stress oxidation condition using H_2_O_2_. However, the oxidation process resulted in single product. This product was purified on silica gel column chromatography and subjected to detailed spectral analysis in comparison with the original drug. Both ^1^H- and ^13^C-NMR data (Table 6) revealed that protons and carbons chemical shifts are closely similar from positions 1–21 (Fig. 1). These data indicated that no changes occurred in this part of the drug. The two overlapped methylene carbons assigned for C-23 and C-27 were *upfield* shifted from δ_c_ 45.87 in SLD to 40.84 ppm in oxidation product. The proton signals correlated to these carbons as indicated by HSQC experiment were changed from broad singlet at δ_H_ 3.10 in SLD to well resolved signals at δ_H_ 3.40 and 3.63 ppm. Similarly, the methylene signals of positions 24 and 26 were shifted from δ_H_ 2.50 (bs), δ_C_ 53.68 ppm in SLD to δ_H_ 3.21 (overl), 3.39 (t, *J* = 11.0) and δ_C_ 65.07 ppm in the oxidation product (Figs. 5,6). Another dramatic change was observed in the chemical shifts of the methyl signals at position 28. The carbon signal *downfield* shifted from δ_C_ 45.64 ppm in SLD to δ_C_ 60.61 ppm in oxidation product. In the ^1^H-NMR, the corresponding methyl signals showed *downfield* shift to δ_H_ 3.20 ppm in the oxidation product compared to δ_H_ 2.27 ppm in SLD (Figs. 5,6). The recorded differences in the NMR data were diagnostic for the conversion of SLD to its N-oxide derivatives at the nitrogen atom of position 25^29,30^. The proposed structure for **2** as SLD N-oxide was further confirmed by HR-ESIMS in both positive and negative modes. A quasi molecular ion was observed at *m*/*z* 491.2067 (cal. 491.2077), in addition to the dimeric ion at *m*/*z* 981.4066 (cal. 981.4075). In the negative mode [M-1]^+^ was observed at m/z 489.1926 (cal. 489.1920) supporting the molecular C_22_H_30_N_6_O_5_S formula for SLD N-oxide [4-((4-ethoxy-3-(1-methyl-7-oxo-3-propyl-4,7-dihydro-1H-pyrazolo[4,3-d]pyrimidin-5-yl)phenyl)sulfonyl)-1-methylpiperazine 1-oxide] (Figs. 7,8).

SLD belong to tertiary amines family and metabolised by the liver enzymes to the N-dealkylation or N-oxide derivatives^31^. The N-oxide and N-dealkylation derivatives are specified in the EP monograph of SLD as impurity B and impurity F respectively^32^. SLD impurity B was quantified in 15 sildenafil citrate generic tablets from international markets obtained via the Internet^33^. The use of SLD can produce adverse reactions such as flushing, headache, nasal congestion and heartburn^34^. The transformation of tertiary amines into N-oxides resulted in generally less toxic derivatives^35^. The adverse effects of SLD occur when the drug reaches its plasma peaks leading to dose frequency and duration limitations. SLD N-oxide was prepared by reaction of SLD with *meta*-chloroperbenzoic acid and proposed as a prodrug. When administered SLD N-oxide undergo liver transformation into SLD itself and the active metabolite N-desmethyl SLD. This process can provide the advantages of prolonged duration of action and less sharp peak plasma concentration. Blunted peak plasma level helps in reducing the side effects of the drug^36^.

In the present study, the efficacy of SLD N-oxide for improving sexual behaviour of male rats in comparison with SLD was conducted for the first time. MF, IF, ML, IL and EL of male animals in the presence of sexually active female rats are important sexual parameters used to assess the aphrodisiac potential of chemical substances and medicinal herbs^37^. Monitoring of the cages showed that the males treated with SLD and SLD N-oxide reacted with rapid movements towards females and showed pre-copulatory actions such as chasing and anogenital sniffing soon after pairing (data not shown).

Oral administration of SLD or SLD N-oxide at 10 mg/kg to male rats showed marked statistically significant (*p* < 0.05) aphrodisiac effect as compared to the NC group (Tables 7,8). Significant (*p* < 0.05) increases in the MF (12.64 ± 0.69) and IF (8.58 ± 0.44) could be clearly seen in SLD N-oxide treated rats as compared to the NC group (6.62 ± 0.52 and 3.20 ± 0.12, respectively). The observed discrepancy in the values of mount frequency and intromission frequency in this investigation implies that not every mount by the male rats climaxed into intromission. The mount frequency and intromission frequency are valuable markers of libido and sexual potency (Yakubu and Atoyebi 2018). Further, intromission frequency can further suggest efficacy of erection and the ease with which ejaculatory reflexes are stimulated^38^. Therefore, marked increase in mount frequency and intromission frequency in male rats treated with SLD N-oxide suggests its ability to support erection process and improve libido^39^. Similarly, SLD increased significantly (*p* < 0.05) the MF and IF compared with rats of the NC group. However, SLD was more potent than the N-oxide derivative (Table 7).

Further, SLD N-oxide produced significant reductions (*p* < 0.05) in the ML (67.87 ± 2.73 s) and IL (127.08 ± 3.74 s), when compared with the NC group (117.35 ± 4.52 s and 221.47 ± 6.73 s, respectively). The mount latency and intromission latency are valuable indices of sexual motivation or arousability^40^. Thus, the reductions in mount latency and intromission latency induced by SLD N-oxide dosing provide evidence of activated sexual motivation and enhanced effectiveness. SLD treated animals showed statistically significant (*p* < 0.05) improvement in mount latency and intromission latency (Table 7).

The PEI is considered as a marker of sexual performance or copulation^41^. It is a valuable evaluation marker for maleness, libido and the rate of recuperation from fatigue following the first series of mating^42^. The reduced postejaculatory interval observed with SLD N-oxide may be due to the increased potency and libido or to the decreased tiredness in the 1st mating series or both. Moreover, premature ejaculation is one of the main reasons of sexual inability. In this study, SLD N-oxide was effective to prolong the EL (Table 8) which is the most important parameter of male sexual behaviour. The capacity of male’s sexual recovery in addition to the aphrodisiac activity can be estimated depending on EL^38^. Accordingly, the prolonged EL of the 1st and 2nd series associated with SLD N-oxide give indication of improved sexual function in male animals^43^. However, SLD N-oxide improvement in the copulatory and the intercopulatory efficiencies were insignificant (*p* < 0.05) compared to those of rats exposed to SLD (Fig. 9).

**Figure 11:**
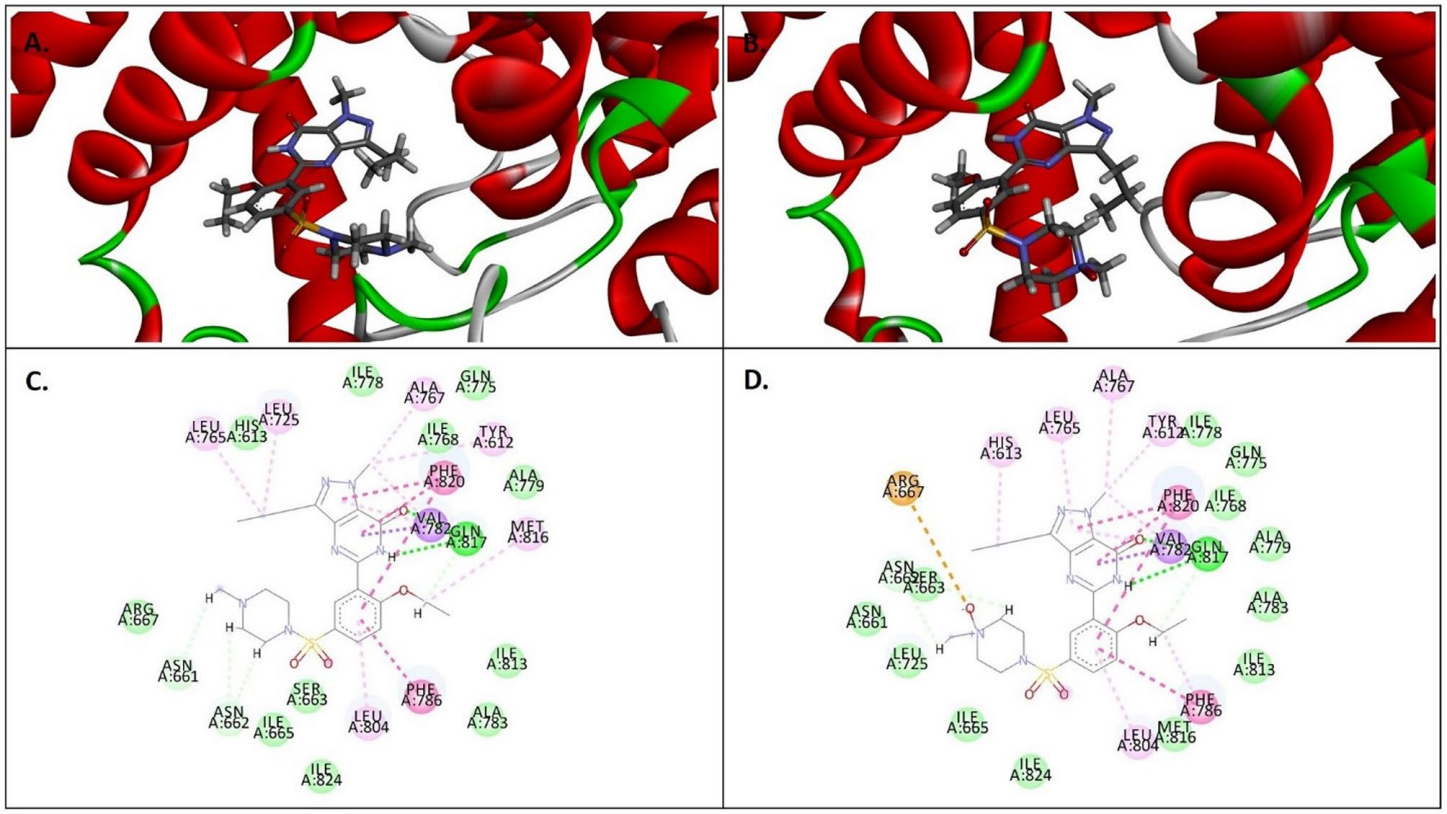
Binding interaction of ligands within the catalytic site of PDE5 enzyme. 3D interaction of (**A**) SLD, (**B**) SLD N-oxide, and 2D interaction of (**C**) SLD, (D) SLD N-oxide. The type of interactions is represented in Discovery studio visualizer by color codes in which hydrogen bond, van der Waals, attractive charges, Pi-Pi, and Pi-Alkyl interactions are depicted in dark green, light green, brown, dark pink, and light pink colours respectively.

Molecular docking was applied to support the results obtained from the animal study. The prepared ligands were subjected to XP docking with PDE5 (PDB ID: 2H42, Resolution 2.30 Å). Both the ligands (SLD and SLD N-oxide) showed strong binding affinity with PDE5. However, the docking score of SLD N-oxide was found to be slightly lower as compared to the parent drug, indicating that SLD is more potent than SLD N-oxide, as shown in Table 9. The 3D and 2D representation of docked conformation of SLD and SLD N-oxide, with PDE5, obtained after XP docking is depicted in Fig. 11. Binding analysis of both SLD and SLD N-oxide in the active site of PDE5 clearly showed the presence of two polar interactions (hydrohen bonds) of the O and NH of pyrazolopyrimidinone group with key amino acid residues Gln817. The pyrazolopyrimidinone moiety and phenyl ring of SLD and SLD N-oxide showed π–π stacking against Phe820 and Phe786 of PDE5, respectively. The oxygen atom of N-oxide of SLD N-oxide showed additional attractive interaction with Arg667. However, the stability of SLD inside the binding pocket was found to be greater as compared to its N-oxide congener, as concluded from the glide XP score. In addition to the above, both ligands were stabilized by several hydrophobic interactions, as depicted in the Fig. 11. These findings support the biological evaluation results where SLD was more active than SLD N-oxide.

**Table 9:** The docking score, *binding energy*, and H-bond interactions of SLD and SLD N-oxide.

Ligands	Glide score	Binding energy (kcal/mol)	H-bond interaction
SLD	− 11.93	− 64.67	O of pyrazolopyrimidinone—Gln817 NH of pyrazolopyrimidinone—Gln817
SLD N-oxide	− 11.71	− 68.25	O of pyrazolopyrimidinone—Gln817 NH of pyrazolopyrimidinone—Gln817

The presented in vivo data supported by docking study indicated that SLD N-oxide has similar effect as SLD. Our finding is also in agreement with the in vitro study indicated that SLD N-oxide has similar selectivity but tenfold less potency than sildenafil. The fact that SLD N-oxide is metabolised in the liver to SLD and the active metabolite N-desmethyl SLD can prolong the duration of action and reduce the adverse effects of the drug^36^.
